# Placental contribution to the endocrinology of gestation and parturition

**DOI:** 10.21451/1984-3143-AR2018-0015

**Published:** 2018-08-03

**Authors:** Gerhard Schuler, Rainer Fürbass, Karl Klisch

**Affiliations:** 1 Veterinary Clinic for Obstetrics, Gynecology and Andrology, Faculty of Veterinary Medicine, Justus Liebig University, Giessen, Germany; 2 Leibniz Institute for Farm Animal Biology (FBN), Dummerstorf, Germany; 3 Institute of Veterinary Anatomy, Vetsuisse Faculty, University of Zurich, Zurich, Switzerland

**Keywords:** gonadotrophins, placenta, placental lactogen, relaxin, steroids.

## Abstract

In addition to many other functions, the placenta is a source of a vast number of autocrine, paracrine and endocrine factors. However, the spectrum of placental regulatory factors, their concentrations, gestational profiles and roles may differ considerably even between phylogenetically closely related species. Depending on the species, placental regulatory factors of a broad range of molecule classes have been found including (glyco-)proteins, peptides, steroids and prostaglandins. Local placental regulatory factors are especially important for the dialogue between the fetal and the maternal compartment immediately at the feto-maternal borderline and for the control of growth, differentiation and functions of the placenta itself. Moreover, placental hormones in a proper sense may also have effects in more remote targets within the maternal compartment, serving functions such as pregnancy-specific adaptations of maternal circulation, provision of hemotrophe to the fetus or the development and function of the mammary gland. Functions of placental hormones in the fetus proper are less clear but may be especially important before the establishment of a functional fetal endocrine system and near term within the highly species-specific networks of signals preparing and initiating parturition. This review takes a comparative view on the situation in different domestic animals focusing on ruminants and on placental hormones occurring at significant concentrations in the maternal circulation.

## Introduction

There is probably no other organ which shows such a structural diversity comparable to that of the placenta (Leiser and Kaufmann, 1994; Wildman *et al*., 2006). However, its functions are in general widely similar between species. The placenta anchors the fetus in the maternal uterus, induces the local immunotolerance preventing rejection of the fetal allograft, provides oxygen and nutrients originating from the maternal compartment and disposes fetal waste products. Moreover, the placenta is a rich source of signal molecules which may have important effects in the maternal or fetal compartment including the placenta itself. Although the overall functions of placental signaling molecules are in general widely conserved between species, the occurrence of individual placental messenger substances and their specific roles may differ significantly between species. The production of numerous signaling molecules in placental tissues is commonly summarized as the placental endocrine function. Endocrine effects in a strict sense are exerted by molecules which are produced by specialized glands and released into the systemic circulation to reach their mostly remote target cells, where they activate specific receptors. However, it is very obvious that the concept of a classical endocrine factor holds true only for a minority of placental signaling molecules. The majority act as local regulators of growth, differentiation and functions via para-, auto-, juxta- or intracrine mechanisms in the placenta itself or in the adjacent endometrium. Moreover, a certain signaling molecule may exert its effects in different types of target cells by more than only one of the above- mentioned mechanisms (e.g. sex steroids or members of the prolactin/growth hormone family). Typically, in postnatal life the release of hormones is regulated by feed-back mechanisms. However, in many cases it is unclear or unknown whether the expression of a certain placental signaling molecule is regulated or just follows a genetically determined program. Depending on the species, profiles of several placental hormones continuously increase in the maternal circulation starting at a specific stage of gestation (e.g. progesterone in sheep, estrogens in domestic ruminants, relaxin in cat, dog and horse, placental lactogen in sheep and goat) and thus obviously reflect to a considerable extent the gain in placental mass. However, important factors which influence placental endocrine function are stress such as nutrient restriction or hypoxia mediated by an increased exposure of the placenta to glucocorticoids (Fowden and Forhead, 2009), and the signals targeting the placenta during the initiation of parturition (see section: The initiation of parturition: the placenta as a target and source of endocrine signals). Under pathological conditions, placental hormone production may be altered in cases of impaired pregnancy including fetal abnormalities, intoxications or placentitis (Hoffmann *et al*., 1996; Ryan *et al*., 2001, 2009).

Due to the large variety of placental signaling molecules and considerable species specific peculiarities, a detailed review on placental endocrine function in a larger number of species would clearly go beyond the scope of this article. Thus, here we focus predominantly on domestic ruminants (cow, sheep, goat) and those regulatory factors which have been measured at significant concentrations in fetal or maternal blood. According to the specific expertise of the authors special emphasis is placed on placental steroidogenesis.

## The placenta as an endocrine organ during pregnancy

### Basic features of the ruminant placenta

**Figure 1:**
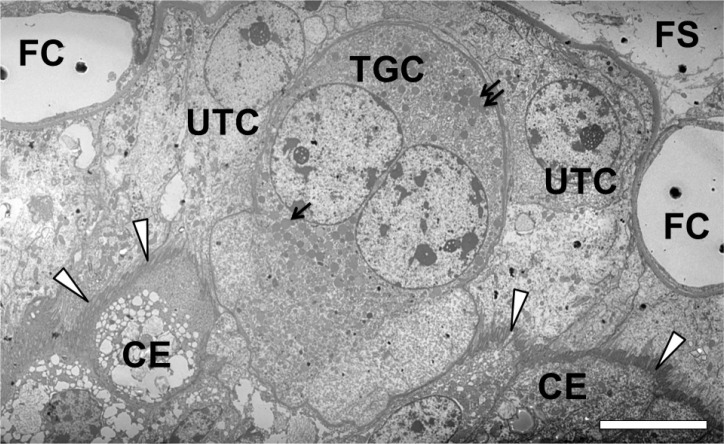
Mature bovine binucleate trophoblast giant cell (TGC) invading the caruncular epithelium (CE; lower part of the micrograph). The TGC is surrounded by several uninucleate trophoblast cells (UTCs). In the TGC cytoplasm numerous secretory granules (small arrows) are visible which are released into the maternal compartment after fusion of a TGC with an individual caruncular epithelial cell. In the secretory granules different signaling molecules have been detected, e.g. placental lactogen and prolactin-related protein-1. The feto-maternal borderline is labeled with arrowheads. FS: fetal stroma; FC: fetal capillaries. Gestation day 150; Bar = 10 μm.

In the ruminant placenta the intimate feto- maternal contact is restricted to multiple discrete structures named placentomes. These are formed during placentation by interactions of the chorion with preformed placentation sites of the endometrium, whose number and placement varies considerably between ruminant species (Hradecky *et al*., 1988). Placentomes are composed of fetal chorionic villi (cotyledon) and the maternal caruncle. In the placentomes fetal villi arising from the chorionic plate of the cotyledon interdigitate with a corresponding system of widely ramified caruncular crypts. Based on initial histomorphological studies the bovine trophoblast was described to be composed of uninucleated cells (UTCs) and larger binucleated cells (BNCs). Later it was realized that in the bovine trophoblast throughout gestation terminally differentiated trophoblast giant cells (TGCs) differentiate continuously from UTCs. The TGCs, which generally possess two octaploid nuclei, become binucleate and subsequently polyploid by a series of acytokinetic mitoses and several stages of TGC development can be observed, which differ in size, location within the trophoblast epithelium and presence of cytoplasmic granules (Klisch *et al*., 1999). As during bovine TGC differentiation binucleated intermediates with lower ploidy levels occur, in this article the term TGC refers to mature trophoblast giant cells. TGCs may fuse with cells of the maternal caruncular epithelium. This fusion leads to short-living trinucleate feto- maternal hybrid cells in cattle and buffaloes (Cavalho *et al*., 2006) and to larger syncytial plaques in sheep and goat (Wooding, 1992). The function of this fusion is thought to be the exocytotic release of fetal derived mediator substances into the maternal compartment (Fig. 1). Due to this fusion the ruminant placental barrier is now classified as synepitheliochorial (Wooding and Wathes, 1980; Wooding, 1992; Klisch *et al*., 1999; Carvalho *et al*., 2006).

### Signals from the periimplantation trophoblast

Already prior to implantation and placentation in many species the trophoblast is a source of signals essential for the establishment and progression of pregnancy with partially considerable differences between individual species, especially with regard to the maternal recognition of pregnancy. In polyoestric spontaneously ovulating species generally a specific signal is necessary to prolong luteal function, which may be luteotrophic (e.g. humans) or antiluteolytic (e.g. domestic ruminants, pig, horse; Spencer *et al*., 2004a; Bazer, 2015). In many species the identity of this/these signal/s or their way of action are still largely unclear or completely unknown. In domestic ruminants trophoblast-derived interferon Tau (IFT) has been identified as the crucial factor for the maintenance of luteal function during early pregnancy. By suppressing endometrial expression of oxytocin receptors, IFT interrupts a positive feedback loop between corpus luteum and endometrium, which would otherwise lead to the exposition of the corpus luteum to luteolytic prostaglandin F2α of endometrial origin (Spencer and Hansen, 2015; Hansen *et al*., 2017). Different from polyoestric species in the monoestric dog a specific luteotrophic or antiluteolytic signal during early gestation is obviously unnecessary as the luteal phase in cyclic bitches lasts longer than gestational length (Kowalewski *et al*., 2015). A different situation is also present in species with induced ovulation (e.g. rabbit, cat, ferret, camelids). During early pregnancy, in addition to ensuring progesterone supply, numerous other essential processes must be initiated and maintained. For example, nutrient supply by the endometrium, endometrial receptivity for implantation, endometrial differentiation, onset of placentation and local immunotolerance, are induced and controlled by a complex network of signals involving the fetal and maternal compartment (embryo-maternal dialogue). Here again, on the fetal side the trophoblast must be considered as the predominant source of signals. Concerning the embryo-maternal dialogue during early pregnancy the reader is referred to excellent and comprehensive reviews (e.g. Bazer *et al*., 2010; Mathew *et al*., 2016).

### The placental endocrine function

#### Placental steroidogenesis

With respect to the regulation of reproductive functions, sex steroids (progestogens, androgens, estrogens) are considered as a prominent class of hormones. However, they are also involved in the regulation of numerous processes outside the reproductive system (Camacho-Arroyo and Montor, 2012), which could also be important in the mother or the growing fetus during pregnancy. Steroidogenic activity has been found in the placenta of many but not all mammalian species, with the dog being an example for a species without any detectable placental steroidogenesis (Hoffmann *et al*., 1994; Nishiyama *et al*., 1999). Research in placental steroidogenesis has mainly focused on the production of progesterone and estrogens and, to a lesser extent, of androgens. However, it is rather likely that bioactive steroids other than classical estrogens, androgens and progestogens may be produced in steroidogenic placentae and may exert important functions during pregnancy.

#### Progesterone and other bioactive progestogens

Progesterone is commonly considered indispensable for mammalian pregnancy, as it is involved in the control of numerous essential pregnancy-related functions including endometrial differentiation, myometrial quiescence, closure of the cervix and local immunotolerance in the pregnant uterus (Chwalisz and Garfield, 1997; Spencer *et al*., 2004b; Arck *et al*., 2007). Initially, the corpus luteum is the source of progesterone in all mammals. In some species the corpus luteum remains the only relevant source of progesterone throughout gestation with minimal (e.g. goat, pig) or no (dog) placental contribution, whereas in other species the placenta adopts this role after a species-specific time of gestation (luteo-placental shift; e.g. in sheep, horse, man; Meyer, 1994; Mitchell and Taggart, 2009). With regard to the relevance of luteal vs. placental progesterone the cow has an intermediate position. Throughout gestation the bovine placenta contributes - if at all - only to a minor extent to the maternal progesterone levels which are predominantly of luteal origin. However, placental progesterone is generally able to maintain pregnancy when luteal progesterone supply is eliminated between day 180 until day 240 by application of luteolytic prostaglandins or surgical removal of the ovaries. After this period, treated cows may immediately abort, exhibit a shortened gestational length or may continue pregnancy until normal term (Estergreen *et al*., 1967; Day, 1977; Johnson *et al*., 1981). Because of these observations bovine placental progesterone synthesis was considered to be a transient phenomenon. However, observations of undiminished placental progesterone tissue concentrations and 3β-hydroxysteroid dehydrogenase activities until term (Tsumagari *et al*., 1994) point to an increased demand of progesterone during late gestation rather than a decrease in placental progesterone synthesis. The role of placental progesterone in species with predominantly luteal progesterone synthesis throughout gestation remains unclear but it may be important for the generation of high local concentration at the feto-maternal interface, which could be necessary for some concentration-dependent progesterone effects (Hansen, 1998). Otherwise, placental progesterone could merely be an intermediate or side product arising from the synthesis of other steroids, especially estrogens. From an evolutionary point of view it is tempting to speculate whether there was an evolutionary pressure for placental progesterone to allow for longer gestation, or whether placental steroidogenesis initially served other functions and subsequently enabled the reduction of luteal lifespan. Commonly, progesterone is considered as the universal master hormone of pregnancy. However, early observations of unusually low or undetectable progesterone levels in individual species (horse, zebra, rock hyrax, elephant) challenged the concept of progesterone as the sole physiological progestogen (Conley and Reynolds, 2014). For the horse it was shown that 5α-dihydroprogesterone circulating at high concentration after the massive onset of placental steroidogenesis is a potent progestogen with a bioactivity comparable to progesterone. It is likely that a systematic investigation into the occurrence of structurally related steroids during pregnancy will result in the discovery of other bioactive progestogens (Scholz *et al*., 2014). The above-mentioned essential functions imply that during pregnancy the maternal compartment is the predominant target of progesterone. Depending on the species, progesterone could also serve as a local regulatory factor in the placenta itself as suggested by the detection of progesterone receptors in the human (Oh *et al*., 2005) or equine chorion (Abd-Elnaeim *et al*., 2009). However, no classical nuclear progesterone receptors were detectable in the fetal part of bovine placentomes (Schuler *et al*., 1999) or in the canine placenta (Vermeirsch *et al*., 2000). Similar to estrogens, on a molecular level the view on the spectrum of possible progesterone effects has become very complex due to the existence of more than one classical nuclear receptor isoform, the existence of membrane-bound receptors and other nonclassical modes of signalling (Garg *et al*., 2017).

#### Placental estrogens

In many mammalian species, especially in primates and ungulates, the placenta produces significant amounts of estrogens. However, observations made so far with respect to the type of estrogens formed, concentrations, gestational profiles in maternal blood and synthetic pathways point to significant species differences. Different from follicular steroidogenesis with estradiol-17β generally being the sole relevant estrogen identified so far, during pregnancy frequently other estrogens are quantitatively dominating in maternal blood. In many ungulate species estrone is the major estrogen and sulfonated forms frequently exceed by far the concentrations of their free counterparts in maternal blood (e.g. sheep: Nathanielsz *et al*., 1982; horse: Hoffmann *et al*., 1996; cattle: Hoffmann *et al*., 1997; llama, alpaca: Aba *et al*., 1998). Moreover, during human (estriol) and equine pregnancy (equilin, equilenin), the formation of species specific placental estrogens is observed (Raeside, 2017). In humans (Loriaux *et al*., 1972; De Hertogh *et al*., 1975), domestic ruminants (Nathanielsz *et al*., 1982; Hoffmann *et al*., 1997) and camelids (Aba *et al*., 1998) maternal estrogen concentrations increase steadily during gestation, whereas horse and donkey mares exhibit a pronounced peak around midgestation (Hoffmann *et al*., 1996; 2014; Crisci *et al*., 2014). The bovine placenta is capable of producing estrogen autonomously from cholesterol (Schuler *et al*., 2008). In contrast, the human and equine placentae depend on the provision of C19 precursors due to a lack of significant CYP17A1 expression. In these species the essential precursors for placental estrogen production are provided by the maternal and fetal adrenal (humans) or fetal gonads (horse), respectively (Raeside, 2017). This interdependence of fetus and placenta for pregnancy associated steroidogenesis led to the term feto-placental unit (Diczfalusy, 1964).

Despite many observations on the biological roles of placental estrogens, definite information is still sparse and their functions may differ considerably between species. It has been suggested that during primate pregnancy placental estrogens have numerous functions such as trophoblast differentiation, autoregulation of placental steroidogenesis, regulation of the maternal cardiovascular system, utero-placental blood flow, placental neovascularization and mammary gland development (Pepe and Albrecht, 1995). However, the dramatic decrease of maternal estrogen levels in cases of placental steroid sulfatase deficiency generally resulted in an only mild impairment of fetal maturity and process of labor, whereas fetal development and placental progesterone production seemed normal. Nevertheless, maternal estrogen levels still remained clearly above basal level and may have been considerably higher locally in placental tissue. Thus, the absence of severe clinical symptoms does not necessarily mean that placental estrogens are unimportant for the above-mentioned functions (Lykkesfelt *et al*., 1984; Pepe and Albrecht 1995). To elucidate the role of placental estrogens in the mare, Pashen and Allen (1979) gonadectomized four equine fetuses between days 197 and 253 of pregnancy, which induced an immediate fall in maternal estrogen levels. The foals were born lighter and their musculature was less developed compared to sham-gonadectomized controls. In a different experimental approach Esteller- Vico *et al*. (2017) applied the aromatase (CYP19A1) inhibitor letrozole to pregnant mares starting on day 240 until parturition. This treatment suppressed maternal estrogen levels by approximately 90% compared to untreated controls but had no effect on uterine artery hemodynamics, normal placental development, maintenance of pregnancy, or neonatal viability. However, neonates from letrozole-treated mares had significantly lower birth weights than controls, pointing to a role of placental estrogens in fetal growth which is not mediated through regulation of uterine blood flow. As discussed above for humans, other roles of placental estrogens may have remained undetected as suppression of placental estrogen production was incomplete. Janowski *et al*. (1996) applied the estrogen receptor antagonist tamoxifen to pregnant cows starting on day 240 until parturition to block the effects of placental estrogens. Animals of the treatment group had significantly lower progesterone concentrations between days -9 to -2 before parturition, but no effects were observed on gestational length, calving, neonatal viability, incidence of placental retention and placental histomorphology. Again, also in this study effects of placental estrogens may have been missed as it remained unclear whether the blockage of estrogen receptors was complete. On a molecular level the effects of placental estrogens are difficult to estimate. Different estrogens may differ substantially in their binding affinities to classical nuclear estrogen receptors (ESR) or may differentially bind to the two ESR paralogues, ESR1 and -2. Weak estrogens act as agonists in the absence of potent ESR ligands, whereas they may have antagonistic effects in the presence of potent ESR ligands. Moreover, the effects of estrogens may be differentially modulated by the specific cellular context, especially by the presence of ESR coactivators or - repressors. Eventually functions mediated by membrane bound receptors and other non-classical signaling pathways also must be taken into account (Zhu *et al*., 2006; Cheskis *et al*., 2007). Another factor contributing to the complexity of estrogenic effects during pregnancy is metabolism, especially the local fine tuning of estrogenic activity by sulfonation of bioactive estrogens and the hydrolysis of estrogen sulfates (Mueller *et al*., 2015; see also section: Placental estrogens - observations from the cow). Commonly, the maternal compartment (uterus, birth canal, mammary gland) is considered as the predominant target of placental estrogens. However, the localization of estrogen receptors in the chorion of various species (e.g. human: Bukovsky *et al*., 2003a, b; cow: Schuler *et al*., 2005; horse: Abd-Elnaeim *et al*., 2009) clearly points to an involvement of placental estrogen in the control of placental differentiation and functions (see also section: Placental estrogens - observations from the cow).

#### Placental estrogens - observations from the cow

In cows the role of placental estrogens has been predominantly seen in the preparation of the birth canal for parturition, in the myometrial excitability at term and in mammary gland development during late gestation. However, maternal estrogen levels start to increase significantly between days 120-150 (Hoffmann *et al*., 1997), and in fetal fluids significant estrogen concentrations have been measured as early as day 30 (Eley *et al*., 1979). Moreover, CYP19A1 transcripts were detected in bovine blastocysts as early as day 7 after insemination, which were exclusively produced from the placenta-specific promoter P1.1 (Fürbass, 2018; Leibniz Institute for Farm Animal Biology, Dummerstorf, Germany; unpublished data). These observations indicate that during bovine gestation estrogens are produced in the conceptus/placenta starting practically from the very beginning of gestation until term. Apart from late gestation in cows the roles of placental estrogens are still unclear and no definite information is available on their effects on a molecular level. In an attempt to identify possible local target cells of placental estrogens, the expression of ESRs was characterized in bovine placentomes by immunohistochemistry between day 150 and term. ESR1 expression was exclusively detected in the maternal part of the placentomes (Schuler *et al*., 2002), where it was especially prominent in caruncular epithelial cells (CECs). As indicated by the proliferation marker Ki67, CECs exhibit a continuously high proliferation, even during late gestation when placentomal growth is minimal. This suggests that proliferation in CECs clearly exceeds the demand resulting from placentomal growth and remodeling (Schuler *et al*., 2000). Light microscopic observations indicate that numerous cells exhibiting features of apoptosis exfoliate from the caruncular epithelium and are phagocytosed in the trophoblast. Thus, in addition to molecules brought by the maternal blood and crossing the placental barrier by diffusion or specific transport mechanisms (hemotrophe), in bovine placentomes degenerating CECs may be an additional important source of nutrients. According to this concept bovine caruncles can be regarded as modified holocrine glands which are colonized by resorbing chorionic villi. Possibly, placental estrogens are an important stimulator of the high proliferation observed in CECs (Schuler *et al*., 2000). Different from ESR1, ESR2 was expressed in various cell types both in the fetal and maternal part of placentomes (Schuler *et al*., 2005). Interestingly ESR2 was significantly up-regulated during the process of TGC differentiation. Evidence for a possible role of placental estrogens in TGC differentiation also comes from the localization of steroidogenic enzymes. The production of estrogens from cholesterol requires the activities of CYP11A1, CYP17A1, HSD3B1 and CYP19A1. Whereas in the trophoblast expression of CYP11A1 and CYP17A1 have been exclusively observed in UTCs, CYP19A1 expression was undetectable in UTCs but was found to increase steadily after the entry of UTCs into TGC differentiation reaching maximal levels in mature and invasive TGCs. The expression pattern of HSD3B1 in bovine trophoblast cells is less clear. However, there is evidence from *in situ* hybridization that its expression is up-regulated during TGC differentiation (Schuler *et al*., 2008). These observations suggest that in bovine trophoblast cells the expression of ESR2 and steroidogenic enzymes is coupled to the process of TGC differentiation and the expression of steroidogenic enzymes is up-regulated in differentiating TGCs concomitant with the availability of their specific substrates. However, the concept of placental steroids as intracrine regulators of TGC differentiation is challenged by the observation that between D180 and late gestation the expression of CYP17A1 is largely restricted to the trophoblast of chorionic stem villi (Schuler *et al*., 2006b), whereas TGC differentiation obviously occurs at any localization within the chorionic villous tree. A role of estrogens in the control of trophoblast cell differentiation has also been suggested in the human placenta (Bukovsky *et al*., 2003a, b).

In domestic ruminants including the cow sulfonated estrogens clearly dominate over free forms throughout gestation except for the immediate pre- and intrapartal period (Hoffmann *et al*., 1997). In bovine gestation, expression patterns of CYP19A1 and of the estrogen specific sulfotransferase SULT1E1 in placentomal tissue provide clear evidence that placental estrogens are to a significant extent sulfonated in the trophoblast immediately after their production in TGC. Intriguingly, in the bovine trophoblast SULT1E1- mRNA was predominantly detected in TGCs, whereas the corresponding protein was virtually exclusively found in UTCs (Khatri *et al*., 2013; Polei *et al*., 2014). Sulfonation of steroids abolishes their interaction with nuclear receptors. Moreover, as sulfonation of steroids markedly increases their polarity, different from lipophilic free steroids they are practically incapable of crossing biological membranes by passive diffusion, which considerably reduces their distribution volume. Thus, traditionally sulfonation of steroids has been primarily regarded as a mechanism leading to their inactivation and facilitating their elimination. On the other hand, sulfonated steroids commonly circulate at significantly higher concentrations compared to their free counterparts, and may (re-)enter the pool of free steroids by the activity of steroid sulfatase (STS). Thus, sulfonated steroids are now increasingly considered as substrates for the local production of bioactive steroids in specific target tissue (sulfatase pathway of steroidogenesis), with their cellular uptake by steroid sulfate transporters and hydrolysis being potential additional levels for the local control of activity (Mueller *et al* 2015). In bovine placentomes a high expression of STS was found in the caruncular epithelium. Moreover, the expression of significant levels of mRNA encoding the steroid sulfate transporter SLC10A6 were also detected in caruncles (Greven *et al*., 2007; Greven, 2008). In conclusion, the expression of CYP19A1, ESRs, SULT1E1, STS and SLC10A6 in close proximity to each other points to a role of placental estrogens as local regulators in bovine placentomes and indicates that their effects are tightly controlled by local mechanisms. However, definite information on their roles and mode of action are still missing.

#### Androgens

Androgens in a strict sense are defined as steroids promoting male secondary sex characteristics. However, the term androgen is commonly also used for their metabolites or C19 steroids such as androstenedione, dehydroepiandrosterone (DHEA) or sulfonated DHEA (DHEAS) which are devoid of any noteworthy activity at the androgen receptor but may serve as precursors for the synthesis of bioactive androgens. It is clear that in species exhibiting placental estrogen production aromatizable C19-steroids must be available as precursors. Due to a lack of considerable CYP17A1 expression, the human and equine placentae depend on the provision of C19-steroids from extraplacental sources (Raeside *et al*., 2017), whereas placentae in other species such as the cow (Schuler *et al*., 1994, 2006a) or the sheep (Mason *et al*., 1989; Gyomorey *et al*., 2000) are capable of efficiently converting C21-steroids into C19-precursors for the production of estrogens. However, as bioactive androgens could disturb sex differentiation in female fetuses or cause virilization in mothers, their unrestricted transfer in relevant amounts into the fetal or maternal circulation must be prevented. In humans one mechanism for androgen inactivation is obviously aromatization (Bulun, 2014). However, the detection of androgen receptors in the steroidogenic placentae of different species, such as humans (Uzelac *et al*., 2010), cattle (Khatri *et al*., 2013), horse (Davis *et al*., 2017) or pig (Wieciech *et al*., 2013) indicates that placental androgens may be more than just precursors for estrogen synthesis. However, the mere presence of androgen receptors in placental tissue does not necessarily imply a function of androgens, as sex steroid receptors may also exert steroid-independent activities due to constitutionally active transcription activation functions of their N-terminal domain or cross-talk with other signaling pathways (Davey and Grossmann, 2016). A completely different situation in comparison to primates or ungulates is present in mouse and rat pregnancy, where placental C19-steroids serve as precursors for the production of estrogens in the ovary, which play an important role in the maintenance of luteal function (Jackson and Albrecht, 1985).

#### Chorionic gonadotrophins

The pituitary gonadotropins luteinizing hormone (LH) and follicle stimulating hormone (FSH) play an outstanding role in the hormonal regulation of gonadal function. Together with thyroid stimulating hormone (TSH), they belong to the family of glycoprotein hormones and are composed of a common α-subunit and a hormone-specific β-subunit. Depending on the species, LH-related glycoproteins are also expressed in the trophoblast. For a long time, chorionic gonadotrophin (CG) expression has only been reported in equids and primates (Cahoreau *et al*., 2015). However, more recently the expression of LH-like substances has been described in a bottlenose dolphin placenta (Watanabe *et al*., 2007). Human CG is composed of the common α-subunit of the glycoprotein hormone family and a specific β_CG_-subunit, which is highly similar to the human β_LH_-subunit but is generated from one of several separate β_CG_-genes. As mentioned above, during early pregnancy hCG from the blastocyst is an essential signal for the maternal recognition of pregnancy as it stimulates the maintenance of luteal function via the binding to luteal LH-receptors until the placenta takes over the role as the relevant source of progesterone. Moreover, the involvement of hCG in other functions during human pregnancy has been identified such as endometrial angiogenesis, quiescence of the myometrium, maternal intrauterine immunotolerance and control of syncytiotrophoblast formation (Perrier d'Hauterive *et al*., 2007; Fournier *et al*., 2015). The CG biology in horses is clearly different from CG biology in humans. The β-subunit of equine CG (eCG) is a product of the *β_LH_* -gene. However, the eCG molecule differs significantly from equine LH in that it is intensely and differentially glycosylated. In contrast to hCG, expression of eCG starts clearly after the maternal recognition of pregnancy (day 35-36). In the otherwise epitheliochorial equine placenta, eCG expression is restricted to a specialized trophoblast subpopulation which invades the maternal endometrium to form discrete structures named endometrial cups. By its strong and long-lasting LH activity in horses, eCG induces the transformation of large follicles, which develop during early pregnancy under the influence of pituitary FSH, into accessory corpora lutea (Murphy and Martinuk, 1991; Allen, 2001; Antczak *et al*., 2013).

#### Pregnancy-specific members of the prolactin and growth hormone family

Growth hormone (GH, somatotropin) and prolactin (PRL) are structurally-related signaling molecules which have evolved from a common precursor. Their receptors (GHR, PRLR) are also structurally related. Whereas in most mammals GH and PRL are evolutionarily conserved, in anthropoid primates, ruminants and muroid rodents either GH, PRL or both have undergone considerable evolutionary change and GH- (primates) or PRL- (rodents, ruminants) gene derivatives are expressed in the trophoblast (Gootwine, 2004; Soares, 2004; Haig, 2008). Moreover, in the elephant placenta the production of an immunoreactive PRL has been described in the trophoblast (Yamamoto *et al*., 2012), whose characterization on a molecular level is still pending. In primates, ruminants and rodents depending on the individual species lineage and gene, the placental GH- or PRL paralogues may exert their effects via the GHR, PRLR, GHR/PRLR dimers or may have non-classical actions (Gootwine, 2004; Soares, 2004; Haig, 2008). The nomenclature of GH/PRL family members is partly confusing. Depending on the individual molecules, the PRL family members in rodents and ruminants have been given a variety of names such as placental lactogen (PL), PRL-like proteins (PLPs), PRL-related proteins (PRPs) and proliferin (PLF). However, the PRL-derived genes encoding ruminant and rodent PLs and PRPs are not orthologous. GH-family members occurring in primates are referred to as PL, GH-variant (GH-V) and chorionic somatomammotropins (CS; Soares *et al*., 2007).

In ruminants both GH- and PRL-encoding genes have undergone considerable evolution. Duplication of the PRL gene has been shown in all well- studied ruminants, leading to the formation of a cluster consisting of PRL, PL and PRP genes. Most ruminant species including cattle possess a single GH-like gene, which despite the absence of gene duplication has also undergone accelerated evolution. However, domestic sheep and goat have been found to be polymorphic for a GH-duplication (GH1/GH2-N and GH2-Z). Whereas in the bovine placenta there is no evidence for GH expression, in the ovine placenta GH was found to be expressed between days 35-75. In ovine haplotypes with two GH genes, one gene was expressed in the pituitary (GH2-N) and the other one in the placenta (GH2-Z). In addition to a role in the control of pregnancy-specific uterine gland differentiation and functions, GH transiently expressed in the ovine placenta has also been suggested to have effects in the fetal compartment before the onset of GH expression in the fetal pituitary (Gootwine, 2004; Reicher *et al*. 2008).

Ruminant PRL and GH signal through their corresponding receptor, respectively. Ruminant PL (syn.: chorionic somatomammotropin hormone, CSH) acts as agonist at PRLR-homodimers and at PRLR-/GHR- heterodimers, whereas it has antagonistic effects at GHR- homodimers (Gootwine 2004). Expression of bovine PL commences in the trophoblast cells around implantation. After the onset of placentation ruminant PL and PRP- expression is virtually confined to TGCs, where it is strongly up-regulated during TGC differentiation from UTCs and is maintained beyond the migration of TGCs into the caruncular epithelium or fusion of TGCs with CECs (TGC-endometrial heterokaryons). Generally, ruminant PLs have been considered as regulatory factors predominantly involved in the control of uterine and mammary gland development and nutrient delivery from the maternal to the fetal compartment (Gootwine, 2004; Soares, 2004; Haig, 2008). PL-deficient ovine pregnancies generated by the application of the lentiviral-mediated short hairpin RNA technique targeting CSH-mRNA to blastocysts followed by their transfer to recipients resulted in phenotypes consistent with that of intrauterine growth restriction, probably by impairment of placental development beginning early in gestation. Moreover, the results suggested that PL- deficiency impacted fetal liver development and function (Baker *et al*. 2016; Jeckel *et al*. 2018). Consistent with a role in endometrial gland differentiation and function, PRLR expression was found in glandular epithelial cells of the ruminant endometrium. Ovine PL has been shown to bind to endometrial PRLR and stimulate the secretion of uterine milk into the uterus lumen (uterolactation). The stimulatory effect of ruminant PL on uterine histiotrophe delivery is obviously mediated by a paracrine mechanism not requiring PL to enter the maternal systemic circulation (Gootwine, 2004; Soares, 2004; Haig, 2008). However, in pregnant sheep (Handwerger *et al*., 1977; Chan *et al*., 1978) and goats (Currie *et al*., 1990) PL concentrations start to increase substantially during the second trimester, reaching peak levels around 1-2 µg/ml during late gestation suggesting endocrine PL effects in the maternal compartment also beyond uterine tissues. Different from rodents in sheep luteotropic and/or luteoprotective actions of PL could not be demonstrated (Al-Gubory *et al*., 2006).

In contrast to sheep and goat, maternal PL concentrations in cattle were only in the lower ng/ml range throughout gestation (Wallace, 1993). In steroid- primed dairy heifers a mammogenic effect of PL was demonstrated (Byatt *et al*., 1997). As no considerable effect of a PRL inhibitor or a PRL antiserum on bovine luteal function could be demonstrated (Hoffmann *et al*., 1974), like in sheep also in cattle a luteotropic action of PL is unlikely. Concentrations in the bovine fetal circulation were somewhat higher than in the maternal with mean values decreasing from about 25-30 ng/ml in early gestation to 10-20 ng/ml prior to parturition (Holland *et al*., 1997; Alvarez-Oxiley *et al*., 2007), suggesting that bovine PL may have effects in the fetus proper.

Ruminant PLs have been postulated as factors involved in the normal physiological adjustments occurring during pregnancy in concert with other regulators. Observations especially from rodents suggest that activities of PRL paralogues may not be particularly in demand in unimpaired pregnancy but may become important during adaptations to stress (Gootwine, 2004; Soares, 2004; Soares *et al*., 2007; Haig, 2008). Concerning the specific biology of GH or PRL paralogues in anthropoid primates and muroid rodents the reader is referred to the excellent reviews cited here and the references included herein.

**Figure 2:**
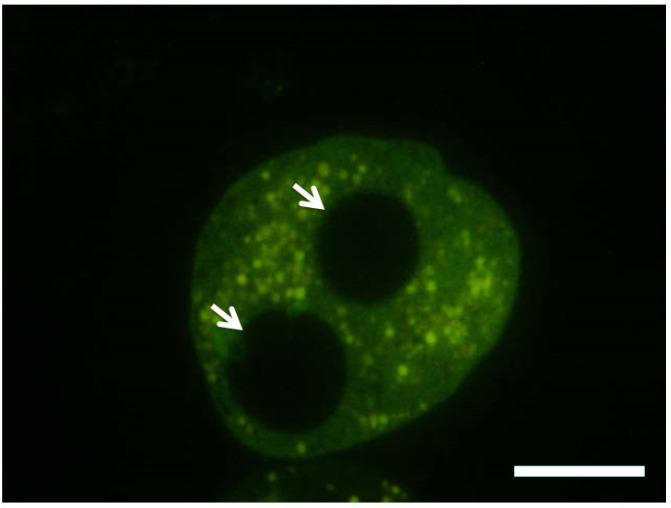
Immunolabelling (green) of prolactin-related protein-I (PRP-I) in secretory granula of a bovine TGC. The two TGC nuclei (arrows) are devoid of immunostaining. Gestation day 110; Bar = 10 μm.

In addition to PL, in ruminants another distinct subfamily of PRL-related placental transcripts has been identified predicting glycoproteins structurally related to PL and PRL, named Prolactin-Related Proteins (PRPs). However, these non-classical members of the PRL/GH family are quite different in amino acid sequence from PL. In cattle on the protein level the expression of only PRP-1 has been confirmed, while mRNAs from more than ten different PRP genes are transcribed. The function of PRP-1 produced in TGCs (Fig. 2) throughout gestation is unclear, as it does not bind to PRL or GH receptors. Results from *in vitro* studies suggest that it may stimulate placentomal angiogenesis (Patel *et al*. 2004; Ushizawa *et al*. 2010).

#### Relaxin/insulin-like family peptides

After the discovery of relaxin by its softening effect on the pubic ligament in virgin guinea pigs (Hisaw, 1926), subsequent research revealed a complex hormone system. In placental mammals it consists of four receptors (RXFP1-4), which are members of the rhodopsin G protein-coupled receptors, and multiple ligands of the relaxin/insulin-like (RLN/INSL) family. Together with the family of the insulin-like growth factors (IGFs) the RLN/INSL family forms the insulin- relaxin superfamily. Evolutionary research provided evidence that the RLN/INSL family traces back to a single progenitor gene in the common ancestor of vertebrates. The diversification of RLN/INSL genes found in contemporary vertebrates resulted from two whole genome duplications in early vertebrate evolution followed by differential additional gain of genes by small scale duplications and gene losses in the individual linages of species (Hoffmann and Opazo, 2011; Yegorov *et al*., 2014). In mammalian species the RLN/INSL gene family generally consists of relaxin 1 (RLN1), relaxin 3 (RLN3) and genes encoding the insulin-like peptides (INSL) 3-6. In an ancestor of humans and great apes a duplication of RLN1 yielded RLNH1 and RLNH2. The latter gene functionally corresponds to RLN1 in other mammals, whereas the function of human RLNH1 is at present widely unclear. RLN/INSL family members are pleiotropic polypeptide hormones which are involved in the regulation of a broad variety of physiological processes. However, RLN1 (human RLNH2), INSL3, INSL4 and INSL6 are clearly associated with reproductive functions in males and females, whereas RLN3 and INSL5 are predominantly associated with the neuroendocrine system and the gut, respectively (Bathgate *et al*., 2013; Anand-Ivell and Ivell, 2014; Arroyo *et al*., 2014). When the term relaxin (RLN) is used in this article the reproductive relaxin (RLN1; human RLNH2) is meant.

Depending on the context RLN may act as a systemic hormone or as a local regulatory factor. In many mammalian species a pregnancy-specific increase of maternal RLN concentrations is observed. Generally, regarding quantities the corpus luteum and/or the placenta are the most important sources. However, their relative contributions to maternal blood levels may differ significantly between species. In a considerable number of species including rabbit, dog, cat, camelids and horse the placenta is the major or sole source of RLN at advanced stages of gestation. Different from the aforementioned species, in humans, rodents and pigs circulating RLN is of luteal origin throughout gestation. However, local effects of placental RLN may also occur in the absence of substantial concentrations in the maternal circulation. A multitude of RLN effects have been described during pregnancy such as decidualization, immunomodulation, quiescence of the myometrium, maternal circulatory adaptations of pregnancy, stimulation of angiogenesis, uterine and vaginal growth, development of the mammary gland and/or nipples. In the prepartal period, RLN has been related to the relaxation of the pubic ligament and the preparation of the maternal birth canal for parturition. However, observations concerning pregnancy-specific roles of RLN point to considerable differences between species (Parry and Vodstrcil, 2007; Bathgate *et al*., 2013; Klein, 2016). The fact that expression of the cognate RLN receptor RXFP1 has been detected in in human (Yamasato *et al*., 2017) and canine trophoblast cells (Nowak *et al*., 2017) indicates that at least in these species RLN may have local effects in the placenta itself. Moreover, the detection of RXFP1 expression in human fetal placental vascular endothelial cells suggests that RLN may have a role in the control of placental perfusion and thus could affect feto-placental growth (Yamasato *et al*., 2017). In addition to direct effects, RLN may also have indirect effects e.g. by the induction of other mediators such as VEGF or nitric oxide (Palejwala *et al*., 2002; Leo *et al*., 2017). In addition to RLN1, also INSL3 was found to serve important reproductive functions in males and females. However, no evidence was found in the literature for a considerable placental expression of INSL3.

Concerning the physiology of the RLN/INSL family domestic ruminants hold a special position among mammals as they do not express RLN1. In cattle the RLN1 gene has obviously been lost during evolution and in goat, sheep and other ruminants only a pseudogene was identified. However, the bovine expresses fully functional receptors for RLN1 and INSL3, RXFP1 and RXFP2, respectively. Thus, it was suggested that in ruminants other members of the RLN/INSL family or non-relaxin ligands could compensate for the missing RLN1 (Dai *et al*., 2017; Malone *et al*., 2017). However, studies to corroborate the concept of a relaxin physiology in ruminants applying porcine relaxin to late pregnant heifers yielded inconsistent results (Musah *et al*., 1986; Bagna *et al*., 1991; Smith *et al*., 1996, 1997).

### The initiation of parturition: the placenta as a target and source of endocrine signals

#### Initiation of parturition in domestic animals

Mammalian parturition is controlled by a complex network of signals involving the fetus, the mother and the placenta. Important processes related to parturition are the withdrawal of progesterone (or progesterone effects), softening of the birth canal, opening of the cervix, increase in the excitability of the myometrium, release of uterotonic substances, rupture of the fetal membranes, expulsion of the fetus and finally the timely release of the placenta. Another process immediately linked to the prepartal endocrine changes is the onset of lactation. However, initiation and control of parturition has been studied in detail only in a limited number of species with the exact sequence or network of events being still widely unknown in most of them. Although some common motifs have been encountered in several species investigated, observations available so far indicate that even between closely related species significant differences may exist (Jenkin and Young, 2004; Mitchell and Taggart, 2009). As the placenta is an important player in the network of signals controlling initiation and the process of parturition, the diversity of its endocrine function significantly contributes to the considerable differences in these signal cascades between species.

According to the current general concept of initiation of parturition in domestic animals, after gradual maturation the fetus reaches a state of readiness for parturition. At this point a rapid mechanism of progesterone withdrawal is activated (Mitchell and Taggart, 2009). The effects induced by the return of progesterone to basal level allow for the final expeditious processes resulting in the onset of myometrial activity and the stretchability of the birth canal eventually accomplishing the expulsion of the fetus(es). One reason for the significant variability between species concerning the underlying chain of events is the difference in progestogen supply during late gestation, thus requiring different mechanisms of prepartal withdrawal. In species with the corpus luteum being the sole or major source of progesterone at the end of gestation (e.g. dog, cat, pig, goat, cattle, mouse, rat and rabbit) luteolysis is considered as a prerequisite for the onset of physiological parturition. In species with only or predominantly placental synthesis of progestogens (sheep, horse), their withdrawal at parturition may come about by the channeling of C21- precursors of progestogen synthesis into a different pathway (Whittle *et al*., 2001; Fowden *et al*., 2008; Mitchell and Taggart, 2009). However, in humans parturition occurs when maternal progesterone levels of placental origin are maximal (Smith *et al*., 2009) and a significant drop of maternal progesterone concentrations is only observed with the release of the placenta. Thus, in humans the concept of a functional progesterone withdrawal was put forward, possibly based on the local withdrawal of progesterone in relevant target tissues by metabolism or changes on the progesterone receptor level or post-receptor signaling mechanisms. However, during late human pregnancy the role of progesterone and the definite mechanisms leading to the cessation of progesterone effects at parturition are still unclear (Zakar and Hertelendy, 2007; Mitchell and Taggart, 2009). Moreover, different from the situation in species with a prepartal progesterone withdrawal initiating rather single-stranded chains of events, in humans it has been suggested that the onset of birth results from a protracted parallel destabilization of pregnancy in several compartments of the fetal-placental-maternal unit synergizing in the transformation of the uterus from a quiescent to a contractile phenotype (modular accumulation of physiological systems; Mitchell and Taggart, 2009). Similar to the situation in humans, also in guinea pigs parturition occurs in the presence of high progesterone levels of placental origin (Illingworth *et al*., 1971). It is evident that a broad variety of placental signaling molecules participates in the feto-maternal dialogue during the period between the initiation of parturition and the timely release of the placenta, including numerous locally acting factors (Streyl *et al*., 2012). For the sake of manageable size also this part of the article is widely limited to placental hormones detectable in significant amounts in the fetal and/or maternal circulation.

#### Initiation of parturition with in species with a prepartal collapse of placental progestogen production (sheep, horse)

**Figure 3:**
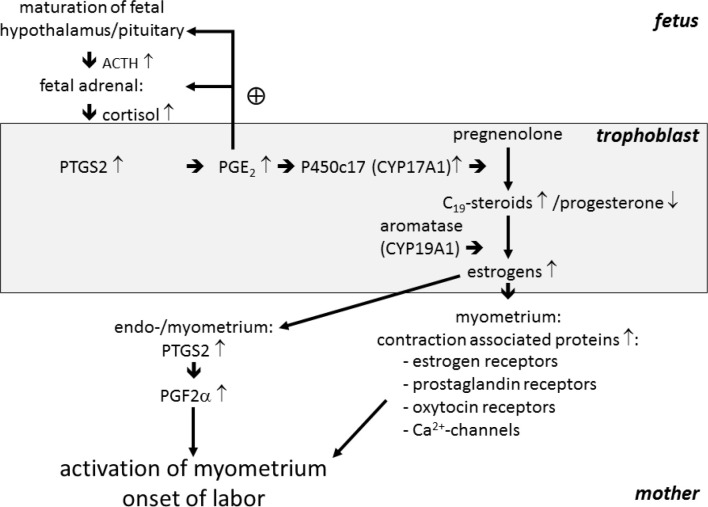
Concept of the endocrine events initiating parturition in the sheep (according to Whittle *et al*., 2001). For description see section: Initiation of parturition with in species with a prepartal collapse of placental progestogen production (sheep, horse) in the text.

**Figure 4:**
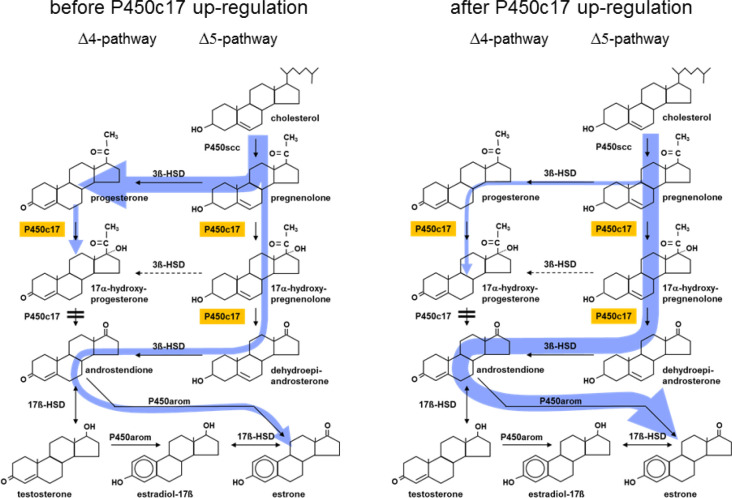
Concept of the prepartal switch in ovine placental steroidogenesis resulting from the up-repulation of CYP17A1 expression in the trophoblast induced by the increase of fetal cortisol levels (see Fig. 3). Due to the minimal lyase activity of ruminant CYP17A1 on the ∆4-pathway, the collapse of placental progesterone synthesis does not result to a noteworthy extent from a direct conversion of progesterone into estrogens but rather from the channeling of the common precursor pregnenolone into the synthesis of estrogens via the ∆5-pathway of steroidogenesis. A considerable up-regulation of placental CYP17A1 expression and activity has also been demonstrated in prepartal cows (Schuler *et al*., 1994; 2006b; Shenavai *et al*., 2012). However, different from the substantial pre- and intrapartal increase of placental estrogens in sheep in cattle a considerable increase in maternal concentrations of placental estrogens does not occur near term. P450scc: cytochrome P450 side-chain-cleavage enzyme (CYP11A1); P450c17: 17α-hydroxylase-C17,20-lyase (CYP17A1); 3ß-HSD: 3ß-hydroxysteroid dehydrogenase-∆5/4-isomerase (HSD3B1); 17ß-HSD: 17ß-hydroxysteroid dehydrogenase (HSD17B); P450arom: aromatase (CYP19A1).

For predominantly historical reasons (Liggins, 1968, 2000) to date the sheep is practically the only species in which a detailed and largely experimentally confirmed concept for the initiation of parturition has been established (Fig. 3), as this species has served for many years as a model to study the physiology of parturition in humans (Whittle *et al*., 2001). As the decisive process triggering physiological initiation of parturition, a progressive maturation and activation of the fetal hypothalamus-pituitary-adrenal (HPA) axis during late gestation has been identified, resulting in a considerably increased cortisol release from the fetal adrenals. The increased fetal cortisol levels stimulate a substantial up-regulation of cyclooxygenase 2 (PTGS2) expression in UTCs, causing an enhanced synthesis of PGE_2_. Besides promotion of the maturation of the fetal HPA axis by a positive feedback loop, placental PGE2 stimulates a pronounced up-regulation of the steroidogenic key enzyme CYP17A1 co-localized with PTGS2 in UTC. The increase in CYP17A1 expression enhances the synthesis of estrogens at the expense of progesterone production (Whittle *et al*., 2000, 2001). However, due to the inefficiency of ruminant CYP17A1 to exert the lyase reaction after 17α-hydroxylation of progesterone, the collapse of placental progesterone synthesis and the concomitant increase of estrogen production does not result from a direct conversion of progesterone to estrogens but rather from an increased channeling of the common precursor pregnenolone into the ∆5-pathway of steroidogenesis (Fig. 4; Mason *et al*., 1989). The prepartal increase in placental estrogen levels is considered as an important signal to the maternal compartment stimulating the up-regulation of contraction-associated proteins (CAPs; e.g. oxytocin receptors, prostaglandin receptors, gap junctions) in the myometrium and the production of uterotonic PGF2α in the endometrium. In concert with effects induced by the concomitant progesterone withdrawal, the prepartal increase in placental estrogens brings about myometrial excitability and stimulation of myometrial activity, finally resulting in the expulsion of the fetus (Whittle *et al*., 2000, 2001). A prepartal decline of placental progestogen levels is also observed in the mare. However, the chain of events leading to parturition in the horse is much less clear. Similar to the sheep, in the equine fetus adrenocorticotropic hormone (ACTH) and cortisol increase significantly during late gestation after maturation of the fetal hypothalamus-pituitary system. However, clearly different from the sheep, the equine placenta lacks a considerable CYP17A1 expression, and placental estrogen synthesis, which depends on C19-precursors provided from the fetal gonads, substantially decreases between midgestation and parturition. Moreover, equine placental progestogen synthesis depends on C21-precursors provided by the fetal compartment with the fetal adrenals generally considered as the relevant source. Thus, for the prepartal collapse of equine placental progestogen synthesis a concept has been put forward according to which the rise of fetal ACTH concentrations during the final phase of gestation stimulates a steep increase of cortisol synthesis in the fetal adrenal at the expense of C21-precursors for placental progestogen synthesis (Thorburn, 1993; Fowden *et al*., 2008). However, the concept of the adrenals as the sole relevant source of fetal C21- precursors for equine placental progestogen synthesis has recently been challenged (Conley, 2016; Legacki *et al*., 2016, 2017).

#### Placenta and prepartal luteolysis

In species with the ovary as the sole or predominant source of progesterone during late gestation, prepartal luteolysis is commonly regarded as the decisive step for the initiation of parturition. Due to the fact that in many polyoestric spontaneously ovulating species PGF2α of endometrial origin has been identified as the luteolytic signal during the ovarian cycle and the observation that in species with luteal progesterone during late gestation parturition may be readily induced with PGF2α or analogues (Kindahl *et al*., 1984; Weems *et al*., 2006), prostaglandins are commonly considered as the luteolytic agent also at term. However, although seemingly obvious, a definite confirmation is still pending and information on the source of the prepartal luteolytic signal is still sparse. Moreover, the regulatory mechanisms for the generation of the luteolytic signal are still widely unclear or unknown.

**Figure 5:**
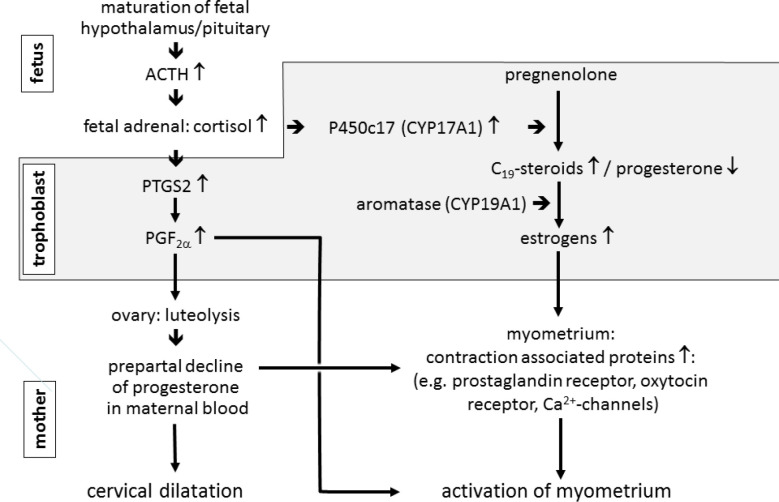
Concept of the initiation of parturition in cattle (according to Shenavai *et al*., 2012). Prepartal endocrine changes similar to the sheep have been confirmed in the prepartal cow (see Fig. 4 and 5). However, different from the late pregnant sheep with the placenta as the only relevant source of progesterone, in cattle the corpus luteum is the predominant source of progesterone throughout gestation and the prepartal decline in maternal progesterone level is clearly associated with luteolysis.

Despite the fundamental difference concerning progesterone supply during late gestation in sheep and cattle, important steps of the signal cascade initiating parturition in the sheep has also been confirmed for the cow (see Fig. 3-5). Observations from pathological prolongation of gestation point to the importance of HPA axis maturation also with respect to termination of bovine pregnancy (Kennedy *et al*., 1957; Buczinski *et al*., 2007; Cornillie *et al*., 2007). Also in the bovine fetus a significant prepartal increase in cortisol concentrations has been demonstrated (Comline *et al*., 1974; Hunter *et al*., 1977), and also in the bovine placenta cortisol stimulates a considerable upregulation of CYP17A1 in UTCs, resulting in the collapse of placental progesterone production (Schuler *et al*., 1994; Shenavai *et al*., 2012). However, as in the late pregnant cow progesterone is mainly of luteal origin, the prepartal decline of progesterone levels observed in maternal circulation closely reflects luteolysis (Hoffmann *et al*., 1979). Thus, the question arises concerning a link between the prepartal changes in bovine placental endocrine function induced by the increase in fetal cortisol and luteolysis. In the ovine pregnant uterus, two different ways have been identified for the production of prostaglandins at the onset of parturition, a cortisol-dependent/estrogen-independent mechanism within the trophoblast leading to the rise in fetal plasma PGE2, and a mechanism stimulated by placental estrogens within the maternal endometrium bringing about the massive release of prostaglandins considered relevant for myometrial activity (Whittle *et al*., 2000). However, during late gestation and at parturition, in the bovine caruncles PTGS2 was undetectable by immunohistochemistry (Schuler *et al*., 2006a), and expression in the intercaruncular endometrium and in the myometrium was low and did not significantly change during the period in question. A strong up-regulation of PTGS2 in the bovine endometrium was detected only on the day after parturition (Fuchs *et al*., 1999; Arosh *et al*., 2004; Schuler *et al*., 2006a; Wehbrink *et al*., 2008). These observations suggest that UTCs, in which PTGS2 is dramatically up-regulated by fetal cortisol around the time of luteolysis, are the major source of luteolytic prostaglandins in the prepartal cow (Fig. 5). This concept is further corroborated by the detection of a significant expression of AKR1B5 in UTCs, an enzyme considered as the relevant prostaglandin F synthase in cyclic cows (Madore *et al*., 2003; Schuler *et al*., 2006a). However, any weak PTGS2 expression in the uterus may not be neglected due to the size of the organ in late pregnant animals. A problem commonly arising in studies concerning the endocrine changes leading to prepartal luteolysis in cows and other species is the exact definition of the time point when luteolysis is initiated, which is commonly based on the time when the decline of maternal progesterone levels becomes obvious. In many cases a precise determination is dubious due to the substantial variability of prepartal progesterone profiles between individual animals and considerable diurnal fluctuations. However, an exact determination of the initiation of luteolysis is crucial for the assessment whether or not an increase in placental or uterine prostaglandin production may be considered as the prepartal luteolytic signal. In prepartal cows, concentrations of PGF2α or of its major metabolite15- keto-13,14-dihydro PGF2α (PGFM) increase substantially concomitant with the onset of labor (Fairclough *et al*., 1975; Edqvist *et al*., 1978; Bosu *et al*., 1984, Meyer *et al*., 1989; Shenavai *et al*., 2012). However, the massive prepartal increase of PGF2α/PGFM levels in the maternal circulation is obviously primarily related to the stimulation of myometrial activity. They may certainly contribute to the final stages of luteolysis, whereas during initial stages, i.e. 36-48 h prior to birth, their rise over basal level is, if at all, only minimal. Moreover the PGFM profile in maternal peripheral blood is distinctly different from luteolysis in cyclic cows (Königsson *et al*., 2001), where prostaglandin spikes of endometrial origin are observed before and during luteolysis (Peterson *et al*., 1975; Kindahl *et al*., 1976; Vighio and Liptrap, 1986). However, marginal PGF2α/PGFM levels during the onset of prepartal luteolysis do not necessarily exclude the role of PGF2α produced in the uterus or placenta as the prepartal luteolytic agent. Similar to a proposed scenario in cyclic cows, PGF2α may reach the ovary predominantly by a local supply system possibly predominantly based on lymphatic vessels. Thus, the local availability of luteolytic prostaglandins at the ovaries may be barely reflected by the profiles showing up in the maternal systemic circulation (Hein *et al*., 1988; Krzymowski and Stefańczyk-Krzymowska, 2008).

The question of the nature and the origin of the prepartal luteolytic signal was also extensively studied in the goat, which exhibits distinct similarities to cattle with respect to estrogen profiles during pregnancy (Sawada *et al*., 1995; Engeland *et al*., 1999; Probo *et al*., 2011), the relative role of luteal vs. placental progesterone (Currie and Thorburn, 1977b; Sheldrick *et al*., 1980) and the prepartal alterations of placental steroidogenesis in response to a prepartal increase of fetal cortisol (Currie and Thorburn, 1977a, b; Flint *et al*., 1978). Results from the studies concerning prepartal luteolysis in goats or their interpretation were partially conflicting. Ford *et al*. (1998, 1999) measured PGF2α or PGFM in late pregnancy in systemic maternal plasma and utero-ovarian venous plasma and did not detect relevant changes in prostaglandin concentrations around the expected time of luteolysis. Therefor they concluded that the results did not support the concept of PGF2α being the principal luteolysin in the pregnant doe at term. However, as in the late pregnant cow (Hein *et al*., 1988), the transport of luteolytic prostaglandins to the ovary by the lymphatic system could not be excluded. Generally, in prepartal goats a gradual decrease of maternal progesterone concentrations starts 3-4 days before parturition. Probo *et al*. (2011) observed a slight increase of maternal PGFM levels starting four days before parturition concomitant with the decline of progesterone. Although the increase of maternal PGFM levels became statistically significant only on the day before parturition, taking into account other observations from the literature (Currie *et al*., 1988) they suggested that luteolysis in prepartal goats is initiated by increasing levels of luteolytic prostaglandins, whereas the onset of their massive production is only possible after the decline of progesterone levels. However, in the literature no information was found which allows identifying a certain cell type in the placenta or uterus as a relevant source of luteolytic prostaglandins in prepartal goats.

A similar situation as described for the cow and the goat is also present in other species which exhibit exclusively or predominantly luteal progesterone supply during late gestation. In these species prepartal luteolysis is considered to be associated with a slight increase of prostaglandins in the maternal blood which precedes their massive rise concomitant with the onset of myometrial activity. Like in the late pregnant cow, a considerable up-regulation of PTGS2 has been found during late gestation in the trophoblast of other species exhibiting prepartal luteolysis such as the dog or cat (Kowalewski *et al*., 2010; Simieniuch *et al*., 2014). However, a definite role of the trophoblast in the generation of the luteolytic signal and possible contributions from other sources in the late pregnant uterus remain to be confirmed. For the prepartal withdrawal of progesterone in the sheep, goat and cow a direct connection with the late gestational rise in fetal cortisol was demonstrated. A rise in fetal cortisol during the prepartal period, which is essential for final maturing processes in several organs, is obviously highly conserved between mammals. However, a direct linkage to the mechanisms initiating parturition is probably unique to ruminant species among Eutherian mammals (Jenkin and Young, 2004).

#### Prostaglandins

Around parturition, across mammalian species prostaglandins are considered as important factors involved in the stimulation of cervical relaxation and myometrial activity, and a massive increase of maternal prostaglandin levels concomitant with the onset of labor is obviously a common trait in mammals (Jenkin and Young, 2004). The key enzyme of inducible prostaglandin synthesis, PTGS2, has been shown to be expressed in the trophoblast of several mammalian species, where it is substantially up-regulated prior to parturition (e.g. dog: Kowalewski *et al*., 2010; cat: Simieniuch *et al*., 2014; cow: Schuler *et al*., 2006b; sheep: McLaren *et al*., 2000). However, the definite contribution of placental prostaglandins vs. prostaglandins from other sources such as the maternal endometrium and myometrium to the massive pre- and intrapartal increase of maternal prostaglandin levels is currently unclear and may differ between species. In the ovine fetus during late gestation PGE2 produced in the trophoblast has been suggested to accelerate maturation of the hypothalamus-pituitary system in response to the rising cortisol levels as a positive feedback mechanism (Whittle *et al*., 2001).

#### Estrogens

Another group of hormones produced in significant amounts in the placentae of many species and considered to be involved in the control of parturition are estrogens. However, similar to pregnancy (see sections: Placental estrogens and Placental estrogens - observations from the cow), the situation of placental estrogens around parturition is very complex, which obscures the understanding of their definite effects. Moreover, information so far available demonstrates significant differences between species.

As mentioned above in the dog, no placental estrogen production is detectable at all. No other estrogen than free estradiol-17β has been described during canine gestation. However, maternal concentrations of estradiol-17β increase only slightly with gestational age but on the average hardly exceed basal levels. As they tend to decline during the last two weeks of gestation with a final drop concomitant with prepartal luteolysis, they are obviously of luteal origin (Hoffmann *et al*., 1994). Thus, in the bitch a significant role of estrogens for parturition related processes is rather unlikely, and the question arises which other factors in the dog serve the roles exerted by placental estrogens in ungulates or primates. In domestic animals placental estrogens are commonly considered as important factors which, prior to parturition, stimulate the softening of the birth canal and induce myometrial excitability and the release of uterotonic prostaglandins. However, a substantial increase in bioactive free estrogens in maternal blood during the immediate prepartal period has only been found in a rather limited number of species such as the sheep (Challis, 1971; Tsang, 1974) or goat (Sawada *et al*., 1995; Probo *et al*., 2011). In other species such as cattle (Hoffmann *et al*., 1997; Shenavai *et al*., 2010, 2012) or camelids (Leon *et al*., 1990; Riveros *et al*., 2009) free estrogens increase, if at all, only moderately throughout late gestation or even decrease markedly prior to parturition as observed in horses and donkeys (Hoffmann *et al*., 1996, 2014). However, as demonstrated in cows (Greven *et al*., 2007; Khatri *et al*., 2011; Polei *et al*., 2014) placental estrogens may be subject to significant metabolism in the pregnant uterus and as the bioactivity of estrogens could be controlled locally in potential target cells (Mueller *et al*., 2015), profiles in maternal blood may not necessarily reflect their local activities. In those ungulates which have been investigated so far during the prepartal period, placental estrogens occur in the maternal circulation predominantly as sulfonated forms and as free estrone, whereas estradiol-17β, the most potent endogenous estrogen, circulates only at much lower concentrations. Moreover, the accuracy of data on estradiol-17ß in pregnant ungulates remains generally unclear, as measurements were mostly performed using immunological methods in the presence of a large excess of other structurally closely related steroids. Thus, even in cases of a weak cross-reactivity of the applied antiserum with other estrogens, published concentrations of estradiol-17β may be considerably overestimated. Nevertheless, observations on estradiol- 17β concentrations in maternal blood indicate that they generally exceed the levels of females at estrus and thus must be considered as biologically relevant. Observations in cattle, sheep and goats provide evidence that in these species the udder contributes significantly to estradiol-17ß levels circulating in the maternal compartment during late gestation and parturition, and CYP19A1 activity was demonstrated in mammary gland tissue *in vitro* (Maule Walker *et al*., 1983; Peaker and Taylor, 1990; Janowski *et al*., 2002). The fact that estradiol-17β concentrations decline rapidly after parturition suggests that the mammary gland could utilize precursors provided by the placenta.

Observations after the experimental elimination of placental estrogen synthesis or blocking of estrogen effects around parturition are rare and partly controversial. Pashen and Allen (1979) gonadectomized equine fetuses between days 197 and 251, which led to an immediate drop of maternal free and conjugated estrogens to basal levels, followed by low estrogen levels throughout the remaining time of pregnancy. In the treated mares parturition started spontaneously. However, uterine contractions were described as weak and inefficient. Correspondingly, the explosive increase of maternal PGFM levels normally occurring in mares during labor was virtually absent. In a different experimental approach Esteller-Vico *et al*. (2017) applied the CYP19A1 inhibitor letrozole to block placental estrogen synthesis in mares throughout the last trimester. However, no cases of dystocia were reported in treated mares, possibly due to the fact that the treatment significantly reduced maternal estrogen levels but blockage of estrogen production was still incomplete. This observation suggests that placental estrogens may have permissive roles, rather than being a regulatory factor and symptoms of deficit may only occur in cases of a virtually complete withdrawal. To elucidate the role of placental estrogens during the initiation of parturition in goats, Currie *et al*. (1976) applied estradiol-17β to late pregnant does. The treatment induced a release of prostaglandin F, regression of corpora lutea, lactogenesis and premature parturition. However, it remains unclear whether the estradiol-17β concentrations in treated animals were in a physiological range and whether the effects induced by the treatment followed the sequence of signals initiating spontaneous parturition.

#### Relaxin

As discussed earlier (see section: Relaxin/insulin-like family peptides), in many mammalian species pregnancy-associated increases of maternal RLN concentrations have been measured with the ovary and/or the placenta being the relevant source(s) depending on the individual species and stage of gestation. On the one hand, together with progesterone, generally RLN is considered as a pregnancy-retaining factor. On the other hand, towards the end of gestation, RLN is considered important for the preparation of the pelvic symphysis and the birth canal for parturition. The softening effect on connective tissue is obviously resulting from remodeling of the extracellular matrix brought about by the increased activity of collagenases (Bathgate *et al*., 2013; Klein, 2016).

## Conclusions

Despite many studies in the field our knowledge on the endocrinology of pregnancy and parturition including the “placental endocrine function” is still very fragmentary or virtually lacking in most species, with the exception of the sheep. Due to significant species specific particularities, concepts put forward based on observations in a certain species may be largely inappropriate in others. The complex interdependencies in the endocrinology of pregnancy and parturition frequently involving different compartments and intricate hormone systems, which are themselves composed of several ligands and receptor types, are difficult to elucidate. Moreover, hormone measurements in the systemic fetal or maternal circulation may not provide the appropriate information about local roles of hormones, which may depend on local mechanisms of transport, activation of inactive precursors or inactivation. Although some aspects may be successfully studied in the refined *in vitro* models nowadays available, for a significant progress in the field animal experiments performed in the respective target species are still considered indispensable. However, experiments in livestock animals are very expensive due to the high costs for the necessary facilities, purchase and keeping of experimental animals or large amounts of compounds applied, e.g. enzyme inhibitors or receptor blockers. Significant progress may be expected from the availability of new or refined analytical methods, e.g. mass spectrometry based methods for the specific simultaneous determination of multiple analytes in blood and tissues, or new efficient technologies to specifically suppress the expression of target genes in the placenta.
